# Longitudinal measurement invariance of memory performance and executive functioning in healthy aging

**DOI:** 10.1371/journal.pone.0204012

**Published:** 2018-09-28

**Authors:** Pedro Silva Moreira, Nadine Santos, Teresa Castanho, Liliana Amorim, Carlos Portugal-Nunes, Nuno Sousa, Patrício Costa

**Affiliations:** 1 Life and Health Sciences Research Institute (ICVS), School of Medicine, University of Minho, Braga, Portugal; 2 ICVS/3B’s, PT Government Associate Laboratory, Braga/Guimarães, Portugal; 3 Clinical Academic Center–Braga, Braga, Portugal; Technion Israel Institute of Technology, ISRAEL

## Abstract

In this work, we examined the longitudinal measurement invariance of a battery composed of distinct cognitive parameters. A sample of 86 individuals (53.5% females; mean age = 65.73), representative of the Portuguese older population, with respect to sex, age and level of education was assessed twice over an average of two years. By means of a confirmatory factor analysis approach, we tested whether a two-factor solution [corresponding to measures of memory performance (MEM) and executive functioning (EXEC)] was reliable over time. Nested models of longitudinal invariance demonstrated the existence of partial strong invariance over time. In other words, this indicates that there is an equivalence of the factorial structure and factor loadings for all items; this was also observed for the item intercepts for all the items, except for one of the items from the EXEC dimension. Stability coefficients revealed high associations between the dimensions over time and that, whereas there was a significant decline of the MEM across time, this was not observed for the EXEC dimension. These findings reveal that changes in MEM and EXEC scores can be attributed to true changes on these constructs, enabling the use of this battery as a reliable method to study cognitive aging.

## Background

The study of cognitive aging has been a topic of great interest for several years among the scientific research, which has been greatly motivated by fact that the world population is progressively living longer. The fact that cognitive aging may interfere with individuals’ quality of life and the ability to make everyday decisions [1] motivates researchers in trying to unveil what are the main variables that may delay the deleterious effects of aging [2]. In this context, both cross-sectional and longitudinal analytical strategies have been implemented. The later approach is of particular relevance, as it allows to capture subject-specific trajectories across time. Nevertheless, there are some limitations associated with this method, considering that cognitive functioning is a multifactorial process that comprises several aspects and that traditional typically focus on individual rather than multiple parameters/dimensions to assess cognitive change.

With the goal of ensuring reliable conclusions concerning repeated measurements it is of upmost relevance to ensure that individual scores between separate times of assessment are representing a similar underlying construct, *i*.*e*., that the follow-up assessment represents the same construct measured at the baseline. More specifically, when relying on the results obtained from dimension reduction techniques, such as principal component analysis or equivalent, it is important to test whether the same factorial structure is observed across groups or distinct measurement intervals. For this purpose, a common approach is to rely on measurement invariance techniques which are widely used to statistically demonstrate that the dimensions are consistent across distinct groups or populations, such as groups or ethnicities [3, 4]. These procedures are implemented by sequentially estimating a series of nested models, in which specific restrictions are progressively established [4]. This approach has also been implemented with the goal of establishing measurement invariance across time–referred as longitudinal measurement invariance [5]. Within the confirmatory factor analysis (CFA) approach, one can test whether factor parameters are similar across time [6].

In this study, with the aim of exploring longitudinal trajectories of executive and memory functions performance during the process of aging, a longitudinal invariance analysis was performed. With this strategy, we intended to avoid simple comparisons between individual tests and, therefore, reducing number of comparisons and, consequently, the likelihood of committing type I errors. Instead, we focused on the creation of two latent variables, referring to executive functioning and memory performance, which were validated using a confirmatory factor analysis (CFA) approach, as previously reported [7].

## Methods

### Participants

Eighty-six community-dwellers [53.5% females; mean age = 65.73 (SD = 8.24)], selected from a larger cohort of 1051 individuals, representative of the Portuguese older population with respect to age and education [7], participated in this study. The study was conducted according to the principles expressed in the Declaration of Helsinki (59^th^ Amendment) and approved by national (Comissão Nacional de Proteção de Dados) and local (Hospital Escala Braga, Braga) ethics committees. A written informed consent was obtained in-person from all the study participants, highlighting: (i) the voluntary nature of the participation on the study, (ii) the right to withdraw at any time, (iii) data confidentiality, (iv) a full description of the study goals, and (v) an overview and explanation of the neuropsychological testing. The assessments were performed individually by a team of experienced examiners.

### Cognitive assessment

A battery of cognitive tests was administered at two time points to test longitudinal effects of aging on global cognition and memory performance. The time between assessments ranged between 18 and 24 months. The battery was comprised of validated tests for the Portuguese population, including: the Mini-Mental State Examination (MMSE) [8], which was used to screen cognitive domains such as orientation, word recall, attention and calculation, language and visual-construction abilities); number of words, colors and interference parameters from the Stroop test [9], which assessed cognitive flexibility and inhibitory control; long-term storage (LTS), consistent-term retrieval (CLTR) and delayed-recall (DR) domains from the Selective Reminding Test (SRT, used to evaluate verbal learning and memory) [10].

Since the tests are assessed with different measurement units, test scores were transformed in order to be expressed in the same scale. In longitudinal data analysis, there are different standardization alternatives, including (i) standardization of repeated measures within individuals, (ii) standardization across individuals within measurement time points and (iii) standardization across individuals across time points, as described in Moeller [11]. These solutions have some limitations, in terms of: examining mean-level differences between individuals, examining mean level changes from one time point to another, and disentangling rank-order and mean-level stability. This is particularly problematic for structural equation modelling procedures, since with the z-score transformation, the information about mean-level changes across time is lost [12]. Possible solutions for handling this issue include z-score standardization for both time-points with baseline as the reference or estimating the proportion of maximum scaling (POMS), according to the formula:

$$POMS=\frac{observed-minimum}{maximum-minimum}$$

Both alternatives allow the covariance matrices to remain suitable for exploratory and confirmatory factor analyses. For our purposes, we used the second alternative, as this can be computed at the individual variable level (*i*.*e*., without the need to adjust for the baseline values). With this approach, each variable varies between 0 (minimum possible value) and 1 (maximum possible value) [11].

Descriptive statistics were obtained for the individual cognitive parameters and intra-item correlations between assessments were estimated. Variables’ distribution was assessed through the analysis of univariate (skewness and kurtosis) and multivariate (Mardia’s test [13]) statistics. Cronbach’s alphas were estimated for assessing the internal consistency of each variable at individual time-points.

### Longitudinal measurement invariance

**Fig 1:**
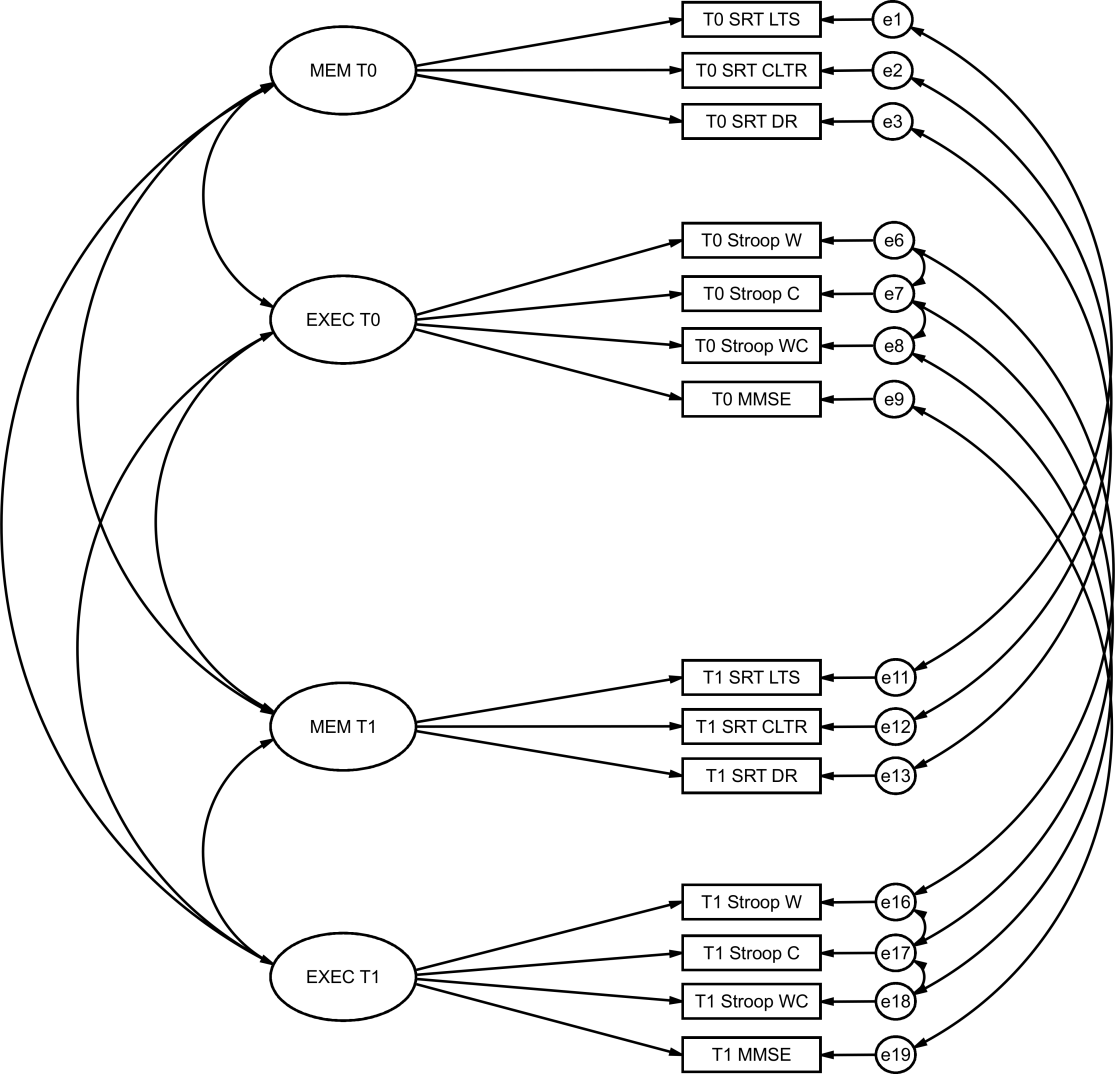
Representation of the model for assessing longitudinal measurement invariance.

To test for longitudinal measurement invariance, four latent variables were defined, corresponding to the measures of executive functioning and memory performance for both the baseline and follow-up periods, respectively (Fig 1). Items corresponding to the same item, measured in both timepoints, were correlated with the goal of accounting for the specific effect associated with each item.

The analytical pipeline was implemented by testing nested models of measurement invariance. In the first step, a baseline model was estimated without any constraints to test whether the factor structure is similar between timepoints–configural invariance. If the configural invariance was assumed, a more restrictive level of invariance was tested, where the factor loadings were constrained to be equal across the two time points–metric invariance, also commonly referred as the weak invariance. The next level of measurement invariance would be the assessment of the equivalence of item intercepts across time intervals–the scalar, or strong invariance–which constitutes a test for systematic response bias across timepoints. Nevertheless, response threshold differences may not reflect biases, but rather expected within-timepoints’ differences, *i*.*e*., if there is a specific expectation that the values of the construct will change between assessments. Indeed, age-related cognitive declines are expected for older individuals. As such, the assessment of this level of invariance is not appropriate and, thus, we did not proceed to its verification. In the absence of a complete longitudinal measurement invariance, partial invariance was tested, by releasing specific constraints on the model (*i*.*e*., factor loadings [14]. The assessment of measurement invariance was assessed by analyzing models’ fit indices. Specifically, each model was assessed, by (i) analyzing its own fit indices and (ii) comparing these properties with the model with lower restrictions. The chi-square statistic (χ^2^) was used to assess goodness-of-fit. Even though this statistic constitutes a fundamental measure for a mathematical comparison of the two matrices, its statistical significance is largely dependent on sample size and may not be an indication of a meaningful discrepancy between the sample and implied covariances. To address this issue, the χ^2^ statistic was complemented with the χ^2^/df ratio, which is aimed to compare the χ^2^ magnitude with the expected values of the sample distribution. The comparative fit index (CFI), the Tucker Lewis index (TLI), the root mean squared error of approximation (RMSEA) and the standardized root mean square residual (SRMR) were used as additional indicators of fit adequacy. To compare the difference of the fit between models, a Satorra-Bentler-scaled chi-square statistic (SB-χ^2^) was used [15]. The assessment of invariance was complemented with the analysis of the difference between other indices. Specifically, following previous recommendations [16], a decrease in CFI of ≥ 0.01 and an increase in RMSEA of ≥ 0.015 was considered unacceptable to establish measurement invariance. The models were conducted using maximum likelihood (ML) estimators. In the presence of multivariate non-normality, a robust ML estimator was used [17]. Given that the establishment of measurement invariance may be influenced by the sample size (which was modest for this statistical procedure, n = 86), Monte Carlo simulation analysis were implemented to assess the power to reject the null hypothesis (H0) of longitudinal invariance. Two sets of data (each with 1.000 datasets of n = 86), based on (i) unconstrained and (ii) constrained models were simulated. Using these simulations, we calculated the percentage of datasets in which the change of specific model fit indices, from the unconstrained to the constrained model, was greater than pre-specified cutoffs (here, a change of 0.01 of the CFI and a significant change of the chi-square statistic). This percentage was the estimated power for the rejection of H0 of longitudinal invariance. We tested the degree to which we would have to constrain the strictest model, so that we would obtain a statistical power (1-β) of .80, corresponding to Cohen’s [18] recommendations (which was suggested base on a β:α ratio of 4:1, considering *i*.*e*., considering typical α and β levels of 0.05 and 0.20, respectively).

With the goal of computing the stability coefficient across time, *i*.*e*., the correlation between factors in distinct timepoints, the factor correlation was estimated, based on the strictest level of longitudinal invariance. For this purpose, the factor variances were constrained to 1 and all the factor loadings were freely estimated. The stability coefficient (correlation between two wave factors) has been previously used as a valid measure to estimate test-retest reliability [19]. Finally, with the goal of analyzing the differences between assessment timepoints in each latent factor, the mean evolution was computed for both dimensions and the statistical significance of the different from baseline to follow-up was assessed.

The R “base” package was used to conduct descriptive bivariate statistics. Measurement invariance analysis was implemented with Mplus [17]. Monte Carlo simulations were implemented with the “simsem” package [20] in R. The code for the statistical analysis is available at the Open Science Framework (https://osf.io/t2vgf).

## Results

Table 1 summarizes the descriptive statistics of the cognitive parameters at baseline and follow-up periods, including the mean, standard deviation, skewness and kurtosis. Participants displayed lower scores on cognitive tests at the follow-up than at the baseline. Univariate skewness [ranging from -1.27 (MMSE_T0_) to 0.86 (SRT CLTR_T1_)] and kurtosis [ranging from -1.06 (SRT DR_T1_) to 1.52 (MMSE_T0_)] were within acceptable limits. The Mardia’s multivariate normality test yielded statistically significant results (Sk_M_ = 793.78, p < .001, K_M_ = 3.13, p = .002). The correlation between items over time is presented in Table 2. As can be observed, the correlation between the same item at different time-points ranged from r = .533 (for the SRT-DR test) to r = .780 (for the Stroop W test).

**Table 1:** Descriptive statistics of MEM and EXEC parameters.

	T0					T1				
	Mean	Median	SD	Skewness	Kurtosis	Mean	Median	SD	Skewness	Kurtosis
SRT LTS	27,16	29,50	13,54	0,06	-0,61	22,36	20,00	13,93	0,60	-0,70
SRT CLTR	16,37	18,00	13,26	0,37	-0,64	15,28	11,00	13,10	0,86	-0,16
SRT DR	5,51	6,00	3,11	-0,10	-0,67	4,24	4,00	3,18	0,15	-1,06
Stroop W	64,83	62,00	21,31	0,10	-0,93	60,12	59,00	24,55	-0,23	0,00
Stroop C	48,35	48,50	16,08	-0,06	-0,03	45,35	45,50	16,84	-0,28	0,05
Stroop WC	28,91	28,00	12,76	0,26	-0,37	27,13	27,00	14,35	0,01	-0,75
MMSE	26,67	27,00	3,29	-1,27	1,52	25,56	26,00	3,65	-1,12	0,74

**Table 2:** Correlation matrix among MEM and EXEC transformed scores for T0 and T1.

	T0—SRT LTS	T0—SRT CLTR	T0—SRT DR	T0—Stroop W	T0—Stroop C	T0—Stroop WC	T0—MMSE	T1—SRT LTS	T1—SRT CLTR	T1—SRT DR	T1—Stroop W	T1—Stroop C	T1—Stroop WC	T1—MMSE
T0—SRT LTS	183.4	159.1	30.9	184.0	154.0	105.3	26.8	112.9	105.8	22.2	229.2	153.5	121.4	29.0
T0—SRT CLTR	.886**	175.9	30.2	171.4	146.0	101.8	27.6	112.1	104.9	20.6	204.0	136.4	108.5	28.4
T0—SRT DR	.735**	.733**	9.7	34.5	28.1	21.6	6.0	25.7	23.5	5.3	43.3	29.9	23.4	6.6
T0—Stroop W	.638**	.607**	.521**	453.9	255.5	180.9	39.3	160.1	147.8	29.6	408.0	237.6	183.9	48.0
T0—Stroop C	.707**	.684**	.562**	.746**	258.7	160.8	30.9	130.2	121.4	22.3	274.0	203.9	164.1	35.9
T0—Stroop WC	.610**	.602**	.544**	.665**	.784**	162.7	20.7	101.8	97.9	16.2	191.4	149.8	133.2	25.1
T0—MMSE	.601**	.632**	.585**	.560**	.584**	.494**	10.8	23.9	22.0	4.9	37.3	26.7	25.3	8.9
T1—SRT LTS	.598**	.607**	.593**	.539**	.581**	.573**	.522**	194.1	175.0	34.5	175.4	147.6	126.3	31.7
T1—SRT CLTR	.596**	.604**	.577**	.529**	.576**	.586**	.511**	.959**	171.7	31.0	162.1	132.9	122.1	28.1
T1—SRT DR	.516**	.490**	.533**	.437**	.436**	.401**	.466**	.779**	.746**	10.1	32.5	26.0	22.1	5.7
T1—Stroop W	.689**	.627**	.568**	.780**	.694**	.611**	.461**	.513**	.504**	.416**	602.7	333.4	252.7	53.2
T1—Stroop C	.673**	.611**	.571**	.662**	.753**	.697**	.482**	.629**	.602**	.487**	.806**	283.6	207.7	35.3
T1—Stroop WC	.624**	.570**	.524**	.601**	.711**	.727**	.536**	.632**	.649**	.486**	.717**	.859**	206.1	30.0
T1—MMSE	.588**	.587**	.584**	.618**	.612**	.540**	.745**	.623**	.588**	.489**	.594**	.574**	.573**	13.3

**p < .001.

The variances of the transformed scores (using proportion of maximum scaling) are represented on the diagonal of the table (light grey); parameters’ covariances are represented on the upper-triangle (dark grey); correlation coefficients are represented on the lower triangle (no shading); underlined coefficients refer to rest-retest reliability.

The results of the cross-sectional CFA model revealed appropriate fit indices for the baseline model (χ^2^_(13)_ = 15.79, p = .261, CFI = .993, TLI = .989, RMSEA = .050, p_(RMSEA)_ = .450). To account for the significant results obtained with the Mardia’s test, robust procedures were conducted to deal with the violation of the multivariate normality assumption. These procedures are less dependent on the assumption of multivariate normality distribution, by producing bias-corrected standard errors and chi-square statistics, given that these deviances are small-to-moderate in magnitude [21]. From the available robust methods, a recent publication demonstrated that the Asparouhov and Muthén maximum likelihood mean- and variance- adjusted (MLMV) produced the most accurate results for small sample sizes and non-normal distributions [22]. Nevertheless, there is a reduced number of studies comparing the appropriateness of each robust procedure with small samples and, to the best of our knowledge, there is a scarcity of published reports comparing different robust methods for longitudinal invariance. Thus, even though we relied on the MLMV method as the primary source for our analytical pipeline, we also compared the results obtained with other robust procedures, including Asparouhov and Muthén mean-adjusted ML (MLR), Satorra and Bentler mean-adjusted ML (MLM).

The results of the longitudinal measurement invariance are presented in Table 3. The unconstrained model exhibited adequate fit properties (χ^2^_(60)_ = 68.78, p = .205; CFI = .988, TLI = .981, SRMR = .042, RMSEA = .041, p_(RMSEA)_ = .603). The comparison between the metric invariance and unconstrained models was statistically significant (SB-χ^2^_(5)_ = 15.61, p = .008), according to the SB-χ^2^ statistic. However, the variation of CFI and RMSEA (CFI = .979, RMSEA = .052) values was within the margin for accepting measurement invariance.

**Table 3:** Model fit indices of nested longitudinal invariance models.

		df	SB-χ2	ΔSB-χ2	p_ΔSB_	CFI	TLI	RMSEA	p_(RMSEA)_	SRMR
MLR	Configural Invariance	60	76.39	—	—	.985	.977	.056	.374	.042
	Metric Invariance	65	91.64	14.48	.013	.976	.966	.069	.174	.061
MLM	Configural Invariance	60	68.98	—	—	.989	.984	.050	.473	.042
	Metric Invariance	65	88.06	14.76	.011	.981	.974	.064	.243	.061
MLMV	Configural Invariance	60	68.78	—	—	.988	.981	.041	.603	.042
	Metric Invariance	65	80.31	15.61	.008	.979	.970	.052	.439	.061

df–degrees of freedom; SB-χ2 –Satorra-Bentler chi-square statistic; CFI–comparative fit index; TLI—Tucker Lewis Index; RMSEA–root mean square error of approximation; SRMR–standardized root mean square residual. All indices are estimated based on robust maximum likelihood estimation.

**Fig 2:**
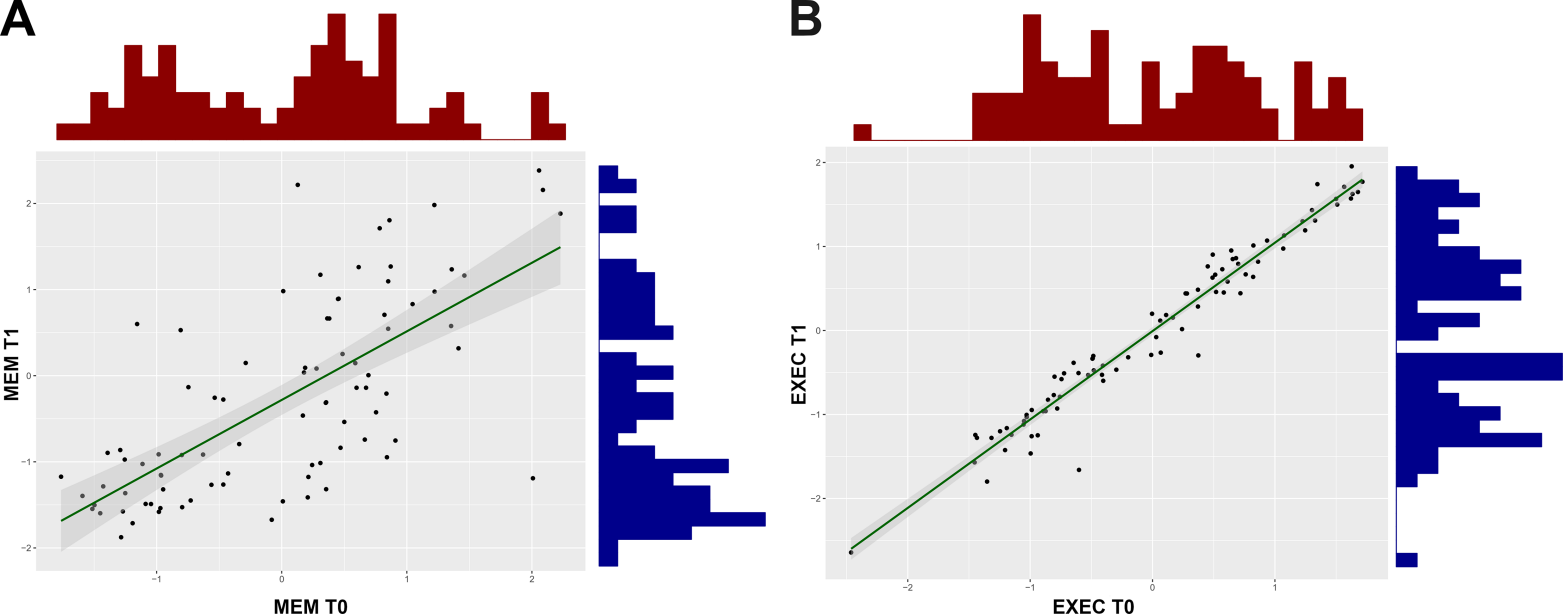
Scatter plots representing the association between timepoints for (A) MEM and (B) EXEC.

This result supports the existence of metric longitudinal invariance. The results from the Monte Carlo simulation study highlighted that a standardized difference of approximately 0.7 in one single loading would have to be observed to reject the null-hypothesis (H0) of longitudinal invariance (with a sample size of n = 86) with a statistical power of 80%. Table 4 displays the variation of the statistical power, according to varying loadings’ standardized differences. While these results demonstrate that large differences need to be observed to detect non-invariance with sufficient statistical power, it would also be relevant to explore whether with large sample sizes one would reject the H0 of longitudinal invariance. To further elucidate on this issue, we implemented a complementary approach in which the real estimates (*i*.*e*., the parameters’ estimates for T0 and T1) were subjected to Monte Carlo simulations with varying sample sizes (from 100 to 1.000 individuals). It was observed that considering the largest tested sample size, the power to reject H0 would be of 51.5% for the chi-square significance (Table 5). The stability coefficient for the global cognition factor between timepoints was computed using the metric invariance model. To achieve this, the factor variances were set to 1 and all the factor loadings for each factor were freely estimated. Cross-sectional associations between latent global cognition and memory demonstrated a larger association at the baseline assessment (r = .845, p < .001) than at the follow-up (r = .710, p < .001) (Fig 2). The difference between these coefficients was statistically significant, as demonstrated by the Fisher r-to-z transformation (z = 2.26, p = .024). The correlations between the same factors measured across time was statistically significant for both the EXEC (r = .942, p < .001) and MEM (r = .663, p < .001), even though the magnitude of association was statistically higher for the first latent factor (z = 6.17, p < .001). These results should not be interpreted as a lack of change from the first to the second timepoints. Instead, they represent a covariation between the scores of MEM and EXEC for T0 and T1, *i*.*e*., the subjects that achieve higher scores at T0 also have higher scores at T1. These associations are visually represented on Fig 2. Finally, considering the inter-factor association between different timepoints, it was observed that while both these correlations between MEM-T1 with EXEC-T2 (r = .824, p < .001) and between EXEC-T1 with MEM-T2 (r = .678, p < .001) are statistically significant, the former is significantly higher than the later (z = 2.21, p = .027).

**Table 4:** Power to reject the null-hypothesis (H0) of longitudinal measurement invariance as a function of varying differences in one factor loading.

Standardized difference	Chi-Square	CFI		
		0.01	0.005	0.002
0.01	0.159	0.085	0.214	0.361
0.02	0.232	0.136	0.31	0.445
0.05	0.646	0.501	0.664	0.754
0.07	0.858	0.74	0.842	0.904

**Table 5:** Estimation of the statistical power to reject the null-hypothesis (H0) of longitudinal measurement invariance, using the obtained estimated for both timepoints, as a function of varying sample size.

Sample Size	Chi-Square
100	0.113
200	0.151
500	0.266
1000	0.515

The estimated factor mean for the follow-up assessment was significantly lower than the baseline values for MEM (ΔM = -.260, p = .010), whereas no statistically significant differences were found for EXEC (ΔM = -.001, p = .984). Thus, the results indicate an equality of latent factor means for EXEC, but not for MEM.

## Discussion

This study explored the longitudinal measurement invariance of a battery of cognitive tests across time in a sample of older individuals. Results revealed that two main dimensions, executive and memory performance, are characterized by longitudinal measurement invariance. In particular, it was observed that there is an equality of the factorial structure and factor loadings across time. From a methodological perspective, it is important to discuss some important limitations of this investigation. Even though we did not detect non-invariance according to previously described approaches in the literature (*i*.*e*., a decrease in CFI of ≥ 0.01 and an increase in RMSEA of ≥ 0.015), the comparison between the metric and configural models originated significant differences of the chi-square statistic. Being the significance of this statistic largely dependent on sample size, both for absolute and difference tests [23], one may argue that obtaining a significant result with a modest sample size would indicate that there is support for the rejection of measurement invariance. While we acknowledge this issue, it is relevant to emphasize that significant changes of the chi-square statistic may arise from trivial discrepancies between the unconstrained and constrained models and from violations of the normality assumption [24]–which was evidenced by the obtained significance of the multivariate Mardia’s test. Furthermore, the results from the Monte Carlo simulations demonstrated that large standardized differences are required for the rejection of measurement invariance with our study’s sample size. Nonetheless, we could also observe that (1) the difference in loadings between timepoints of assessment was below 0.1 standardized units for all the items–which is considered of little importance [25]–and that (2) the actual differences between the two timepoints did not lead to the rejection of measurement invariance with simulated large sample sizes (n = 1.000) with sufficient statistical power. This provides evidence for the stability of the latent measures across time.

These results indicate that the parameters comprising the two dimensions covary across time. From a neurobiological perspective, this suggests the existence of a common basis underlying individuals’ performance on the different parameters comprising each of these dimensions. It is relevant to note that the executive dimension was particularly stable across time, as demonstrated by the absence of statistically significant differences between T0 and T1. On the other hand, memory displays a considerably steeper decay across time, which points towards a dissociable decline of cognitive functioning during the process of aging [1, 26]. In addition, the considerable association between memory performance at T0 and the executive dimension at T1 highlights the relevance of how the actual memory performance may impact the cognitive trajectory during the process of aging.

With these findings, it is demonstrated that using a standardized battery of cognitive tests as an alternative for assessing cognitive evolution in a longitudinal fashion may be a reliable practice. In fact, given that the composition of the latent factors follows a similar structure between separate assessments, this may be of upmost relevance for reducing the amount of comparisons and, consequently the likelihood of committing type I-errors [27, 28]. In sum, with this work we demonstrated the appropriateness of using this battery of cognitive tests to measure two latent constructs, memory and executive functioning. Furthermore, due to the observation of longitudinal invariance at the scalar level, we conclude that these measures can be compared across time [29, 30] as a means to establish growth trajectories during the process of aging.

## Supporting information

S1 Table Individual estimates for all invariance models. (XLSX)
